# Using the International Index of Erectile Function-15 in Comparative Analysis Between Transcutaneous Electrical Nerve Stimulation of the Pudendal Nerve and Low-Level Laser Therapy in the Treatment of Erectile Dysfunction After COVID-19

**DOI:** 10.3390/jcm14228193

**Published:** 2025-11-19

**Authors:** Mustafa Al-Zamil, Natalia G. Kulikova, Denis M. Zalozhnev, Natalia A. Shnayder, Marina M. Petrova, Natalia P. Garganeeva, Natalia G. Zhukova, Olga V. Tutinina, Margarita V. Naprienko, Larisa V. Smekalkina

**Affiliations:** 1 Department of Physiotherapy, Faculty of Continuing Medical Education, Peoples’ Friendship University of Russia, 117198 Moscow, Russia; kulikovang777@mail.ru; 2 Department of Sports Medicine and Medical Rehabilitation, I.M. Sechenov First Moscow State Medical University, 119991 Moscow, Russia; 3 Department of Restorative Medicine and Neurorehabilitation, Medical Dental Institute, 127253 Moscow, Russia; 4 Institute of Personalized Psychiatry and Neurology, V.M. Bekhterev National Medical Research Centre for Psychiatry and Neurology, 192019 Saint Petersburg, Russia; 5 Shared Core Facilities “Molecular and Cell Technologies”, Prof. V.F. Voino-Yasenetsky Krasnoyarsk State Medical University, 660022 Krasnoyarsk, Russia; 6 Department of General Medical Practice and Outpatient Therapy, Siberian State Medical University, 634050 Tomsk, Russia; 7 Department Neurology and Neurosurgery, Siberian State Medical University, 634508 Tomsk, Russia; 8 Department of Outpatient Care and Family Medicine, Prof. V.F. Voino-Yasenetsky Krasnoyarsk State Medical University, 660022 Krasnoyarsk, Russia

**Keywords:** COVID-19, transcutaneous electrical nerve stimulation, TENS, low-level laser therapy, LLLT, 15-question international index of erectile function, IIEF-15, erectile dysfunction, orgasmic dysfunction, sexual desire, intercourse satisfaction, recovery, pudendal nerve

## Abstract

**Background:** Erectile dysfunction (ED) is one of the manifestations of long COVID-19 and in most cases has an endothelial and neurogenic nature. Many experimental and clinical investigations have revealed the high efficacy of transcutaneous electrical nerve stimulation (TENS) of the pudendal nerve and low-level laser therapy (LLLT) in the treatment of ED. **Purpose:** To compare LLLT and TENS, and investigate the dynamics of their efficacy when combined in the treatment of patients with post-COVID-19 ED using the International Index of Erectile Function-15 (IIEF-15). **Materials and Methods:** This interventional, randomized controlled trial enrolled 82 patients with ED following COVID-19. All patients had their first ED diagnosis after COVID-19 within one month of the onset of respiratory symptoms. The duration of patients’ ED was not less than six months, but less than one year. Patients were divided into four groups, one of which received sham LLLT and TENS (n = 20). The remaining patients underwent effective treatment using LLLT (n = 21), TENS (n = 21), and combined LLLT and TENS (n = 20). To study the effectiveness of the treatment, IIEF-15 and an assessment of tactile sensation in the genital area before and after the treatment, as well as 3 months after the end of the treatment, were used. **Results:** Both LLLT and TENS had a significant effect in improving erectile function, of 38% (*p* ≤ 0.01) and 27% (*p* ≤ 0.01), respectively. The improvement in erectile function after LLLT was higher than after TENS by 8.2% (*p* ≤ 0.05), but the combination of these methods exceeds the result of using LLLT alone by 20% (*p* ≤ 0.01). The reduction in hypoesthesia after LLLT did not exceed 17.4% (*p* ≤ 0.05). However, after TENS, the reduction in hypoesthesia reached 48.7% (*p* ≤ 0.01), and with a combination of the two methods, it reached 60.9% (*p* ≤ 0.01). Treatment outcomes in LLLT, TENS, and LLLT + TENS groups were stable for 3 months. **Conclusions:** According to IIEF-15 dynamics, LLLT and TENS are both very beneficial in treatment of post-COVID-19 ED, with LLLT showing a moderately better outcome than TENS. LLLT and TENS were found to have significant positive therapeutic effects on orgasm, sexual desire, and sexual satisfaction, among other aspects of sexual function. Nevertheless, the combination of LLLT and TENS proved to be much more successful in enhancing all IIEF domains, expanding the therapeutic effect spectrum, and improving the TENS effect following LLLT application. Only after TENS did genital hypoesthesia reliably regress, and the effect was amplified when TENS and LLLT were combined.

## 1. Introduction

Once the COVID-19 pandemic was over, it became important and necessary to rethink the specific clinical manifestations of this infection, which took 6.9 million people between March 2020 and May 2023 [1]. Initially, it seemed that during the acute period of the pandemic, respiratory damage was the only critical manifestation of COVID-19. However, in the early stages of the pandemic, many reports published on the extrapulmonary manifestation of COVID-19, such as multisystem venous thromboembolism, as well as acute renal and cardiac failure, which turned out to be the most common causes of mortality and morbidity attributable to this disease [2]. If these complications are caused by pro-inflammatory cytokine upregulation (cytokine storm) [3], then other extrapulmonary manifestations [4,5,6] are associated with autoimmune reactions [7], chronic hypoxia [2], hyperinflammatory [8], and hypercoagulability [9]. Gradually, the term “long COVID-19” began to appear more frequently in the literature [10], associated with the direct penetration of the SARS-CoV-2 virus into extrapulmonary organs through specific receptors, known as Angiotensin-Converting Enzyme 2 (ACE2) [11]. This mechanism underlies the pathology of many organs containing cells with ACE2 receptors [4,5,6,12].

Erectile dysfunction is one of the manifestations of long COVID-19. The exact percentage of patients with post-COVID-19 ED can be found in the literature with highly variable statistics, since in many regions of the world this problem is carefully hidden, as it is assessed by the patients themselves as shameful and humiliating [13]. In addition, given the high latency rates during the first waves of this infection, in many cases physicians did not ask about erectile dysfunction during the clinical examination, and patients were hesitant to discuss it [14]. However, according to some data, the prevalence of this pathology varied between 13.1% and 71.2% [15,16], with most patients (68.1%) developing symptoms of erectile dysfunction within a month after the first clinical signs of COVID-19, and half of them continuing to suffer from ED for 2 years, despite active treatment [17].

Post-COVID-19 ED has a complex polyetiological nature; vascular, neuropathic, endocrine, and psychological mechanisms are involved in its pathogenesis [18]. In many cases, erectile dysfunction is temporary and recovery may occur spontaneously within a few weeks [14]. In such an event, erectile dysfunction occurs due to respiratory failure and impaired oxygenation of the corpora cavernosa and corpus spongiosum in patients with acute hypoxemic respiratory failure [19]. In addition, fatigue and multiple organ failure can be one of the main causes of temporary erectile dysfunction [20]. Other etiological factors are socio-economic in nature, due to the obvious economic consequences of quarantine, emotional stress, and mental engrossment [21].

The most common cause of persistent ED is vascular disorders involving impaired blood flow and outflow in the corpora cavernosa and corpus spongiosum, which are responsible for the mechanisms of penile erection. After penetrating the endothelial cells of the corpora cavernosa and corpus spongiosum via ACE2, the virus interacts with cellular transmembrane serine protease 2, which facilitates the penetration of the virus into cells and accelerates viral replication [17]. Subsequently, endotheliitis and endotheliopathy ultimately lead to erectile dysfunction due to changes in vascular tone, oxidative stress, endothelial–mesenchymal transition, and mitochondrial dysfunction.

The neurotropism of SARS-CoV-2 has been demonstrated in many experimental studies in vivo [22,23] and in vitro [24,25]. Nerve cells of the central and peripheral nervous systems suffer equally due to the penetration of viruses through ACE2. Central sexual regulation may be impaired by SARS-CoV-2-associated hippocampal and neocortical disorders [26]. However, pudendal neuropathy plays a decisive role in the development of erectile dysfunction due to a decrease in the reflexogenic sexual stimulation of neurotransmitters of the cavernous nerve endings. In addition, the development of denervated weakness in the pelvic floor muscles, including the ischiocavernosus and bulbospongiosus muscles, leads to the decreased contraction of these muscles at the base of the corpora cavernosa during erection, which ultimately impedes penile hemodynamics and reduces its rigidity [27,28]. Numerous studies have shown that depressive and anxiety disorders occur in almost all patients with post-COVID-19 ED. However, it is difficult to determine how primary these changes are [17,29,30]. Decreased testosterone levels were found in the majority of patients with post-COVID-19 ED [31]. It is incomprehensible, but the cause of this hormonal deficiency has not yet been established, and it is not known whether it is associated with the suppression of testicular Leydig cells’ secretion [32] or with hypothalamic–pituitary–testicular axis dysfunction [33].

While the American Urological Association and European Association of Urology recommend phosphodiesterase type 5 inhibitors (PDE5 inhibitors) as a first-line treatment for vasculogenic ED [34,35,36], significant drawbacks exist, including a 30–50% non-response rate and frequent discontinuation due to adverse effects [37]. Indeed, the long clinical use of PDE5 has proven its high efficiency in the treatment of ED in many cases. However, simultaneous inhibition of PDE6 can lead to an increase in cGMP levels in both rod and cone photoreceptors. These changes can lead to the dysfunction of photoreceptors and contribute to the development of transient visual symptoms [38]. These disorders in many patients pose a threat to vision and greatly impair comfort and pleasure during sexual intercourse. Less frequently, more serious complications like chorioretinopathy, vessel occlusion, retinal detachment, and optic neuropathy may develop [39,40]. Recently, numerous experimental and clinical studies have shown that platelet-rich plasma, stem cell therapy, and immune system modulation to be emerging regenerative treatments for vascular erectile dysfunction [41]. The therapeutic effect described is achieved through the accumulation of growth factors, stimulating endothelial cells’ proliferation, and extracellular matrix regeneration [42,43,44]. Consequently, the recovery of cavernous nerve fibers and angiogenesis in corpora cavernosa are accelerated [41,45,46,47,48]. While promising, especially for vasculogenic ED, these methods need more research to be applied to post-COVID-related endothelial and neurogenic ED, which may have different underlying mechanisms.

Many experimental and clinical investigations have revealed the high efficacy of transcutaneous electrical nerve stimulation of the pudendal nerve (TENS) [49,50,51] and low-level laser therapy (LLLT) [52,53] in the treatment of ED.

The effectiveness of LLLT in the treatment of ED is explained by the activation of mitochondria by electromagnetic radiation of the laser, which is accompanied by the stimulation and enhancement of ATP production and accumulation in cells. This mechanism promotes the acceleration of metabolic, regenerative, protective, and antioxidant biochemical reactions in endothelial cells [52,54]. One of the main pathophysiological mechanisms of LLLT is the accumulation of intracellular calcium (Ca^2+^) in the cytoplasm by an influx of extracellular calcium into the cytoplasm due to direct modulation of the activity of membrane calcium channels [55,56], as well as due to the transfer of (Ca^2+^) from intracellular depots and the inhibition of calcium sequestration in cellular organelles [57,58]. Moderate accumulation of (Ca^2+^) in the cytoplasm not only protects the endothelium from apoptosis [59] but also stimulates the synthesis and metabolism of nitric oxide (NO) in cells by activating certain isoforms of endothelial nitric oxide synthase [60]. NO is the most important generator mechanism of erectile function, which is characterized by relaxation of the smooth muscles of the endothelial cells of the corpora cavernosa and corpus spongiosum, which leads to penile vasodilation and erection [53,61,62,63]. There are few published studies on the treatment of post-COVID-19 endothelial dysfunction. However, Sergey Moskvin and co-authors reported a high efficiency of LLLT in prevention and treatment of this pathology with a significant reduction in hypoxia after just five procedures [64].

TENS therapy has long been used in the treatment of many diseases of the peripheral and central nervous system [65,66,67]. Its effectiveness has been proven by numerous experimental and clinical studies not only with regard to reducing pain syndrome but also with regard to enhancing regenerative and protective mechanisms [68,69,70,71,72]. TENS indirectly affects penile hemodynamics by stimulating peripheral nerves and passive muscle contractions [73]. However, some studies have shown the effect of TENS on flow-mediated dilation, controlled by endothelial cells, which ultimately contributes to increased blood flow in the vessels and accelerated regeneration in altered tissues, and can be used to reduce the risk of endothelial dysfunction [74]. The effectiveness of TENS in the treatment of ED of various etiologies is determined, first of all, by pudendal nerve recovery [49]. However, supplementary mechanisms have been observed, including a direct reduction in arterial tone and peripheral vascular resistance. This effect is mediated by the stimulation of postganglionic sympathetic efferent fibers, as well as the normalization of sympathovagal regulation [75,76].

To our knowledge, there have been no studies on the effectiveness of LLLT and TENS in treatment of post-COVID-19 ED. Moreover, no comparative analysis of LLLT and TENS has been conducted in the treatment of ED has been conducted at all. Meeting the requirements of the uncovered field, we decided to devote this study to comparing LLLT and TENS and to investigating the dynamics of their efficacy when combined in the treatment of patients with post-COVID-19 ED, using the International Index of Erectile Function-15.

## 2. Materials and Methods

### 2.1. Study Design and Participants

This study is part of scientific program of the Department of Restorative Medicine and Neurorehabilitation of the Russian Medical Dental Institute, “Efficacy of transcutaneous electrical nerve stimulation in the treatment of central and peripheral diseases”, registered on 2 December 2024. The first participant was randomized in our study 4 December 2024. International registration number: ISRCTN47534508—ISRCTN registry, https://doi.org/10.1186/ISRCTN47534508 (accessed on 10 December 2024). The type of study is an interventional single-blind, randomized, four-arm controlled trial.

#### 2.1.1. Randomization of Participants

Eligibility: A total of 345 patients with a suspected diagnosis of post-COVID-19 ED were assessed for eligibility; of these 231 patients did not meet the inclusion criteria and 21 patients refused to participate.

Randomization: The remaining 93 patients were randomized into four groups: 23 patients received sham LLLT and sham TENS (control group), 22 patients passed LLLT (LLLT group), 23 patients underwent TENS (TENS group), and 23 patients were treated by combined LLLT and TENS (LLLT + TENS group).

Completed treatment: The number of patients who completed treatment was 22 in the control group, 21 in the LLLT group, 22 in the TENS group, and 21 in the LLLT + TENS group. During the treatment period, one patient in the control group, two patients in the LLLT group, and two patients in the TENS group withdrew their consent, and one patient in the LLLT group and one patient in the LLLT + TENS group were lost to follow-up.

**Figure 1:**
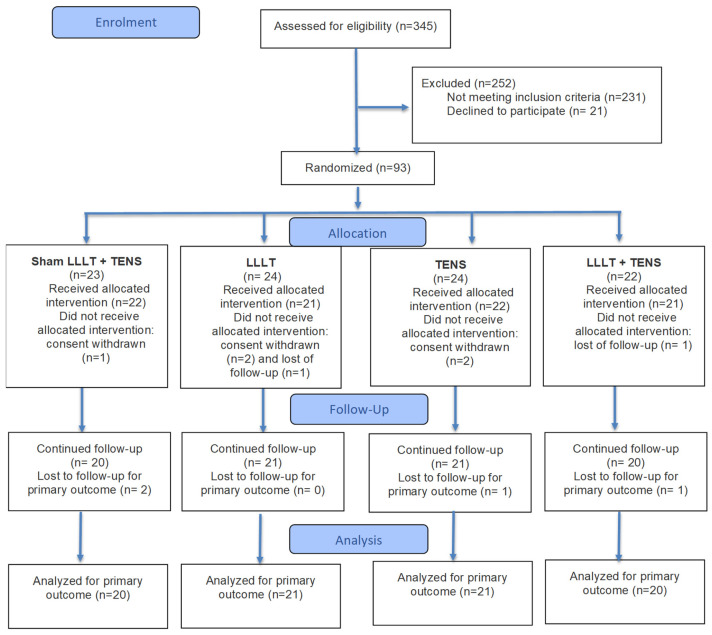
The CONSORT flow diagram. Note: TENS—transcutaneous electrical nerve stimulation; LLLT—low-level laser therapy.

Completed follow-up: In total, 20 patients in the control group, 21 patients in the LLLT group, 21 patients in the TENS group, and 20 patients in the LLLT + TENS group completed three months of post-monitoring. In total, two patients in the control group, one patient in the TENS group, and one patient in the LLLT + TENS group were lost to follow-up (Figure 1).

#### 2.1.2. Participant Selection Criteria

The inclusion criteria were as follows:

1. Europeans;
2. Men;
3. Age—from 21 to 60 years old;
4. ED was first diagnosed after COVID-19, no later than one month after the onset of respiratory symptoms;
5. Erectile dysfunction as a complication of COVID-19 was diagnosed by two urologists.
6. Duration of ED—from 6 months to a year;
7. All patients underwent vascular, nootropic, and vitamin therapy without sufficient effects;
8. Mild or moderate erectile dysfunction with a score in the erectile function domain (EFD) of the International Index of Erectile Function-15 (IIEF-15) greater than 11 and less than 25 points;
9. Hypoesthesia in the penis area from 3 to 7 points;
10. Signed consent to participate.

The exclusion criteria were as follows:

1. Epileptic seizures and other paroxysmal disorders;
2. Mental disorders;
3. Distal polyneuropathy;
4. Central nervous system lesion;
5. Endocrine disorders;
6. Urologic disorders;
7. Peyronie’s disease;
8. Atherosclerosis and structural abnormalities of the penile arteries, internal pudendal arteries, and internal iliac artery;
9. Veno-occlusive dysfunction;
10. Active implantable medical devices.

#### 2.1.3. Consent to Participate

All participants were informed about the purpose and course of the study, the competence of the participating physicians and medical staff, and about similar studies conducted previously to confirm the effectiveness and safety of the treatment and diagnostic methods used. After this, all patients gave their signed consent to participate. Consent for publication was signed after reading the manuscript. Participants and investigators did not receive any compensation for their participation in this study.

#### 2.1.4. Ethics Approval

The approval of the Ethics Committee of the Russian Medical Dental Institute was registered on 22 December 2022, protocol No. 30. The procedures followed the ethical guidelines established in the 1984 Helsinki Declaration and its later updates.

#### 2.1.5. Patient Demographics and Characteristics

The mean age of the participants was 41.4 ± 4.4 years (Table 1). The mean duration of ED after COVID-19 was 8.75 ± 1.2 months. During this period, patients underwent a mean duration of ED pharmacotherapy course of 2.59 ± 0.42 months. The severity of ED by IIEF-15 ranged from 11 to 25 points, with a mean of 15.2 ± 1.1 points. The mandatory condition for inclusion was the presence of hypoesthesia from two to seven points. However, in general, sensory loss in the genital area was not pronounced (mean—3.57 ± 0.65 points).

### 2.2. Sample Size Calculation

In a previous study [77], it was shown that in patients with erectile dysfunction, the EFD of IIEF-15 after the use of TENS improved from 14.1 ± 4.4 to 21 ± 6.0 points, while no significant changes were observed in the control group. To calculate sample size, a sample size calculator in ClinCalc.com was used. The sample size of 19 patients is determined based on a desired power of 95%, a type I error rate of 0.01 and an expected significance level of 0.05.

### 2.3. Clinical Examuination and Outcomes Evaluation

In all patients, COVID-19 was confirmed by medical records and laboratory testing methods. A diagnosis of erectile dysfunction due to COVID-19 complication was made by a urologist based on medical history, ultrasound, and laboratory tests, in line with American Urological Association and European Association of Urology guidlines [34,35,36]. The diagnosis was confirmed by the head of the urology department, who has over 15 years of experience. The examination and assessment of primary and secondary clinical manifestations of ED were carried out three times: before treatment, immediately after treatment, and 3 months after the end of treatment.

#### 2.3.1. Primary Endpoints

##### Erectile Function Domain by IIEF-15

The IIEF-15 is a multidimensional questionnaire for the assessment of ED. The IIEF-15 contains 15 questions covering all aspects of male sexuality. The questionnaire consists of five aspects or domains. The questions of each aspect are not arranged in order. The severity of the disorder in each question is assessed on a five-point scale; a minimum score of zero for questions 1–9 and one for questions 10–15 corresponds to a severe disorder, and maximum score of five points corresponds to the absence of pathology. The severity of the EFD is determined by the sum of the answers to six questions 1, 2, 3, 4, 5, and 15. This domain is the largest and is entirely dedicated to defining erectile dysfunction. In this study, ED was designated as the primary endpoint, while the other four domains (orgasmic function, sexual desire, intercourse satisfaction, and overall satisfaction) were classified as secondary endpoints. The severity of ED was determined by the EFD value and was considered severe with a score of 6 to 10 points, moderate with a score of 11 to 17 points, and mild with a score of 18 to 25 points. At the same time, men with a score of 26 to 30 points were contemplated healthy, without erectile dysfunction [78].

##### Hypoesthesia

Hypoesthesia was determined in the area of innervation of the pudendal nerve, especially in the area of the penis and scrotum, compared with the sensation in the face. Hypesthesia was determined on a ten-point scale. A score of zero points corresponds to the absence of sensory disturbances, and ten points to pronounced sensory disorders.

#### 2.3.2. Secondary Endpoints

##### Orgasmic Function

This is an integral part of the IIEF-15, which reflects the strength of achieving orgasm during sex. The domain level is determined based on the answers to questions 9 and 10. The maximum number of points is ten, the minimum is one point. In our study, before completing the IIEF-15 questionnaire, we explained to patients that the orgasm specified in the questions was an orgasm achieved during penetrative intercourse. This is because different interpretations of the questions may lead to significantly different results.

##### Sexual Desire

The sexual desire domain is a special section of the IIEF-15 on sexual desire, with two items (11 and 12) addressing the level of sexual interest or attraction in men. The maximum number of points is ten and the minimum is one.

##### Sexual Satisfaction

Sexual satisfaction was assessed by defining two domains of the IIEF-15: intercourse satisfaction and overall satisfaction. The intercourse satisfaction domain is a specific aspect of IIEF-15 that focuses on the assessment of the subjective experience and satisfaction derived from sexual intercourse. This domain covers the degree of satisfaction a person receives from various aspects of sexual intercourse, such as the quality of erection, the possibility of penetration, and the ability to maintain an erection to completion. The level of this domain is determined by the sum of points for six, seven, and eight items. The maximum number of points is 15 and the minimum is 3. The overall satisfaction domain is another tool to determine the patient’s satisfaction with sex. This domain includes two questions (13 and 14) regarding satisfaction with sexual experience and sexual life. Thus, general satisfaction is intended to assess sexual health in general. The maximum score for this domain is ten, and the minimum is two.

#### 2.3.3. Exploratory Endpoints

##### Electromyography Changes

Needle electromyography was used to determine the presence of denervation–reinnervation activity in the pelvic floor muscles. The main indication for the use of these studies is confirmation of damage to the pudendal nerve. The second important indication is the assessment of the degree of damage to the pelvic floor muscles adjacent to the base of the corpora cavernosa. Needle electromyography (EMG) of the pelvic floor muscles was performed using a specialized concentric needle on the pubococcygeus muscles bilaterally. Typically, between three and six months following nerve damage, denervation abnormal spontaneous activity, such as fibrillation and positive sharp wave potentials (action potentials of a single denervated muscle fiber), can usually be detected when the muscle is fully relaxed at rest. However, the sphincters are always tonically active, which makes it difficult to detect active denervation changes when simultaneously motor unit potentials (MUPs) are registered. This makes identifying abnormal spontaneous activity during an EMG examination of the pelvic floor muscles challenging. Thus, in our study we focused on identifying and assessing signs of chronic denervation–reinnervation changes, such as polyphasicity, duration prolongation of more than 10 ms, and amplitude increase in more than 1000 μV [79]. Only muscles with noticeable denervation–reinnervation changes were included in the statistical analysis. From 20 recorded MUPs, the percentages of identified polyphasic, extended, and high-amplitude MUPs were calculated.

The EMG examination was carried out using the Neuroexpeditor hardware complex manufactured by MBN, Moscow, Russia, registration number FSR 2010/07889, dated 5 December 2017.

##### Penile Doppler Ultrasound

To exclude patients with vascular disorders associated with atherosclerosis of the penile arteries and their occlusion or stenosis by atherosclerotic plaques, penile Doppler ultrasound (PDU) was used. PDU was performed by measuring systolic and diastolic blood flow in the corporal arteries before and after the intracorporeal administration of a vasoactive drug at a dose of 10–20 μg. The study was conducted using the Siemens ACUSON S1000 device, manufactured by Medical Solutions, Issaquah, WA, USA, registration number RZN 2017/6106, dated 27 April 2018.

### 2.4. Treatment Protocols

#### 2.4.1. Transcutaneous Electrical Nerve Stimulation

**Figure 2:**
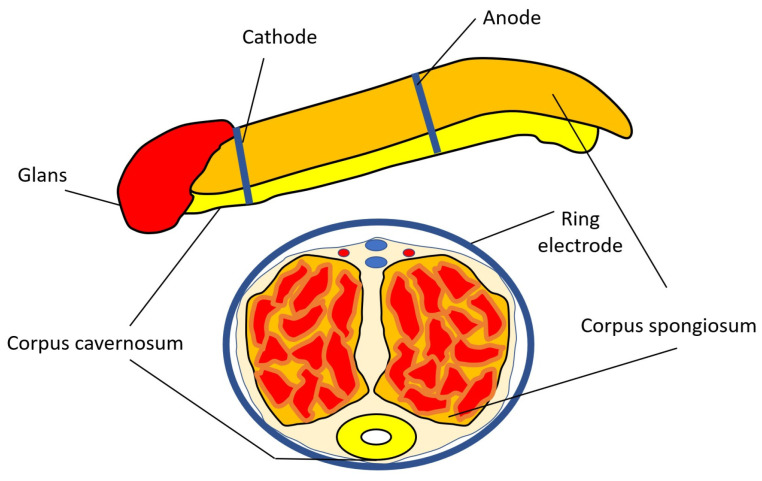
Methodology of applying transcutaneous electrical nerve stimulation to the penis.

A rectangular monopolar current with stable fixation of ring electrodes was used. The cathode was fixed in the proximal part of the penis, the anode, in the distal part (Figure 2). A high-frequency current with a frequency of 50 Hz and a duration of 50 μs was applied for 5 min. Over the next 5 min, low-frequency current with a frequency of 1 Hz and a duration of 200 μs was applied. In the first and second periods, the current amplitude was increased from zero until a comfortable sensory response was achieved. Stimulation according to this method was repeated after a 3 min break. The total number of procedures was 14. Seven procedures were performed every other day, and an additional seven procedures were administered twice a week. The total duration of the course was about 38 days.

TENS therapy was performed using the BTL-4000 smart device (BTL Industries Ltd., Stevenage, UK) with registration number RAN 2020/12648, dated 24 November 2020.

#### 2.4.2. Low-Level Laser Therapy

**Figure 3:**
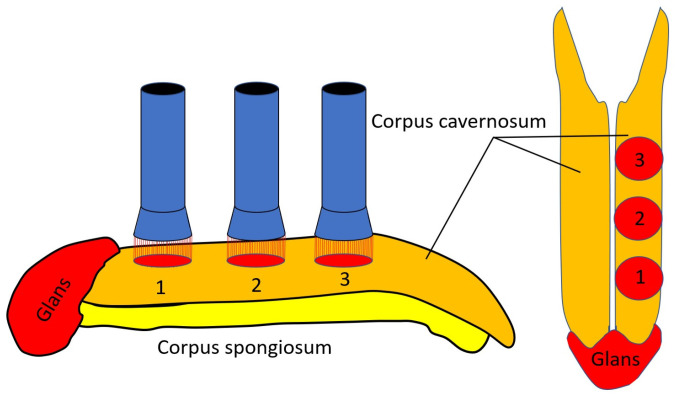
Technique of low-level laser therapy application over the left corpus cavernosum. 1: distal part, 2: middle part, and 3: proximal part.

LLLT was performed using a laser with a wavelength of 904 nm. The light pulses utilized in the therapy had the following characteristics: a duration of 100 ns, a pulse power of 10 W, and power density of 10 W/cm^2^. A frequency of 80 Hz was maintained for the pulses. The LLLT was applied with specific exposure and localization details. The therapy was administered in a total of six zones. These zones were distributed with three in each cavernous corpus. The application sites were localized above the spine of the penis in the proximal, middle, and distal parts (Figure 3). Each zone received an exposure time of 1.5 min. The procedures were repeated seven times every other day and twice a week seven times. The course ran for approximately 38 days.

The procedures were performed using the Lazmik laser physiotherapy device (2 laser channels). The device was manufactured by Scientific and Research Centre MATRIX, Limited Liability Company, Moscow, Russia, registration number RZN 2015/2687, and was dated 25 May 2015.

#### 2.4.3. Combination of Transcutaneous Electrical Nerve Stimulation and Low-Level Laser Therapy

Patients in TENS + LLLT group underwent a combined TENS and LLLT. LLLT was applied according to the method described above. Three minutes after the end of the LLLT session, a TENS session was given for 10 min, according to the scheme mentioned above: 5 min of high-frequency TENS and 5 min of low-frequency TENS. The procedures were performed seven times every other day, and then continued seven times twice a week. The course lasted approximately 38 days.

#### 2.4.4. Sham TENS and LLLT

The patients in the control group received sham TENS + LLLT. Sham LLLT was performed by turning on the device but without emitting light waves. Sham TENS was administered by stimulating the lower abdomen with ineffective electrical impulses at a frequency of 1 Hz, and a duration of 50 μs with a minimal impulse amplitude. The number of procedures was fourteen, which were conducted every other day seven times and twice a week seven times. In total, the course lasted about 38 days.

### 2.5. Statistical Analysis

The interventional factorial, four-arm, single-blind, fixed-block randomization design was used in SPSS for Windows (version 20) software.

In this study, participants were blinded and did not know whether they were receiving the active treatment or a placebo. However, the researcher conducting the study knew which treatment each participant was receiving. This is because in physical therapy, it is impossible to conceal the treatment from the researcher.

The primary endpoints in this research were the IIEF-15 erectile function domain and the degree of genital hypoesthesia. The secondary endpoints were orgasmic function, sexual desire, and sexual satisfaction. To negotiate differences, in baseline values between groups, data transformations, normalization, and outlier limitation were performed. The results of the EMG and ultrasound examination are considered exploratory.

To compare significant differences between groups, the mean (M) and standard deviation (SD) were calculated, followed by Student’s *t*-test and *p*-value. A *p*-value ≤ 0.05 indicates strong evidence against the null hypothesis.

The Shapiro–Wilk test was used to test whether the sample belongs to a normal distribution. The Levene test was used to assess the equality of variances of two or more groups. Bonferroni correction was used to decrease the chances of type I errors when performing multiple hypothesis tests simultaneously. In our study, comparative analysis was conducted only between two groups for one specific symptom: between TENS and LLLT groups, and between LLLT + TENS group and one of the LLLT or TENS groups. Comparisons between two groups do not require the application of the Bonferroni correction. To compare the mean pre-treatment values of the four groups, analysis of variance (ANOVA) was used.

## 3. Results

### 3.1. Primary Clinical Outcomes

#### 3.1.1. Erectile Dysfunction Domain by IIEF-15

Before treatment, the mean level of ED was 15.3 ± 1.2 points, without significant differences between groups (F = 0.22, *p*-value = 0.87).

**Figure 4:**
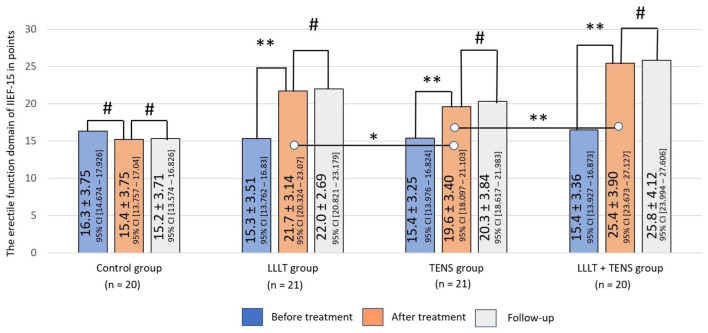
Erectile function dynamics as assessed by the erectile function domain of IIEF-15 in the observed groups. Notes: LLLT—low-level laser therapy, TENS—transcutaneous electrical nerve stimulation, #—*p* > 0.05, *—*p* ≤ 0.05, and **—*p* ≤ 0.01. The black lines highlight the significant differences between the values in the connected columns.

After treatment, no notable changes were observed in the control group. Both groups (LLLT and TENS) reported significant improvements in ED, of 38% (*t* = 6.16, *p*-value = 0.0001, Cohen’s d = 0.85) and 27% (*t* = 4.026, *p*-value = 0.0003, Cohen’s d = 1.26), respectively. Comparing the obtained results, it was found that the improvement in erectile function after LLLT was higher than after TENS, by 8.2% (*t* = 2.03, *p*-value = 0.049, Cohen’s d = 0.64). However, the improvement in erectile function after the combined use of LLLT and TENS was 64.2% (*t* = 8.90, *p*-value = 0.0001, Cohen’s d = 2.73). This result was 20% (*t* = 3.16, *p*-value = 0.003, Cohen’s d = 1.05) higher than after the use of LLLT alone. The treatment outcomes in LLLT, TENS, and LLLT + TENS groups were stable for 3 months (Figure 4).

#### 3.1.2. Hypoesthesia

Hypoesthesia in the area of innervation of the pudendal nerve, and, in particular, in the genital area, was reduced in all patients examined, as this was included in the inclusion criteria for this study. Hypoesthesia prior to treatment was 3.90 ± 0.92 points in the control group, 3.83 ± 1.23 points in LLLT group, 3.9 ± 1.12 points in TENS group, and 3.83 ± 0.912 points in the LLLT + TENS group. No significant differences between groups were recorded (F = 0.32, *p*-value = 0.99).

**Figure 5:**
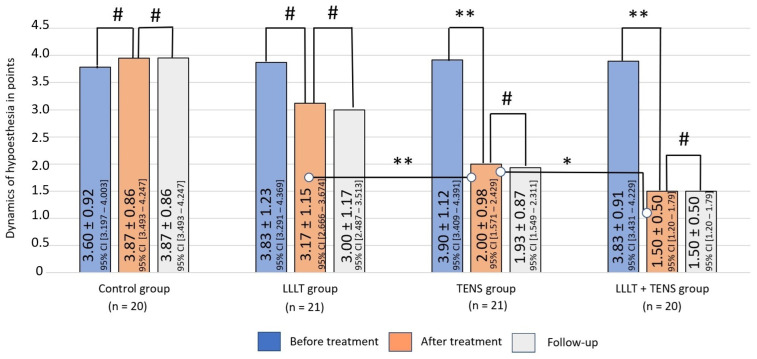
Dynamics of hypesthesia in the study groups. Notes: LLLT—low-level laser therapy, TENS—transcutaneous electrical nerve stimulation, #—*p* > 0.05, *—*p* ≤ 0.05, and **—*p* ≤ 0.01. The black lines highlight the significant differences between the values in the connected columns.

After treatment, a reduction in the severity of hypoesthesia was minimal and insignificant in the LLLT group—17.2% (*t* = 1.76, *p*-value = 0.08, Cohen’s d = 0.55). The mean hypoesthesia percentage decrease was 48.7% (*t* = 6.99, *p*-value = 0.0001, Cohen’s d = 1.80) in the TENS group, and this percentage reduced to 60.9% (*t* = 11.1, *p*-value = 0.0001, Cohen’s d = 3.17) when combining the two LLLT and TENS methods. A comparative analysis between the groups showed that the reduction in hypoesthesia after TENS was 36.9% (*t* = 3.46, *p*-value = 0.0013, Cohen’s d = 1.09) more than after LLLT. With combined use, hypoesthesia decreased by an additional 25% (*t* = 2.032, *p*-value = 0.0491, Cohen’s d = 0.64), compared to with TENS use only, suggesting a synergistic effect of the combined treatment (Figure 5).

No negative dynamics were observed in either group during the post-treatment period.

### 3.2. Secondary Clinical Outcomes

#### 3.2.1. Orgasmic Function

**Figure 6:**
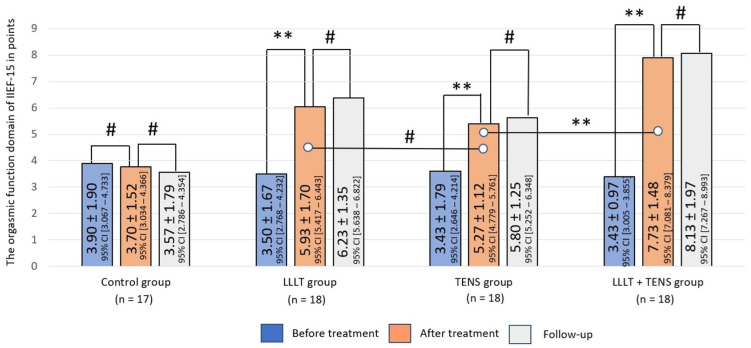
Dynamics of the orgasmic function domain of IIEF-15 in the study groups. Notes—LLLT—low-level laser therapy, TENS—transcutaneous electrical nerve stimulation, #—*p* > 0.05 and **—*p* ≤ 0.01. The black lines highlight the significant differences between the values in the connected columns.

The ability to achieve orgasm during sexual intercourse was reduced in all patients and was around the same in all study groups, with mean score of 3.43 ± 1.2 points (F = 0.388, *p*-value = 0.762). After treatment, improvement was noted in all treatment groups, but not in the control group. The orgasmic function domain value significantly was improved by 69.5% (*t* = 4.32, *p*-value = 0.0001, Cohen’s d = 1.44) after LLLT and by 53.6% (*t* = 3.75, *p*-value = 0.0006, Cohen’s d = 1.25) after TENS (Figure 6). This positive result of LLLT was increased by 30.5% (*t* = 3.38, *p*-value = 0.002, Cohen’s d = 1.12) when LLLT was used in combination with TENS. After completing the treatment, a moderate sustained improvement was observed in the LLLT, TENS, and LLLT + TENS groups of 5.05%, 4.43%, and 5.17%, respectively. However, the reliability of these results is debatable.

#### 3.2.2. Sexual Desire

**Figure 7:**
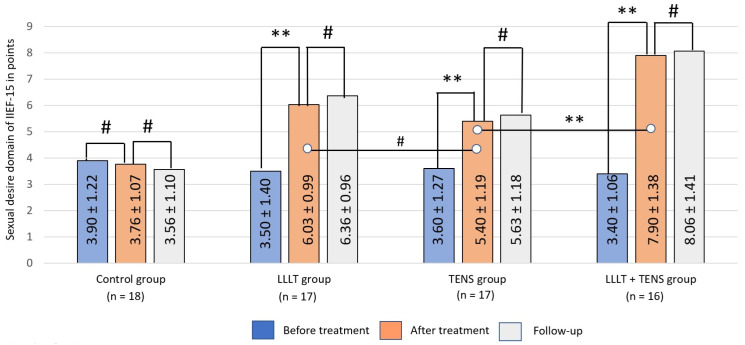
Dynamics of the desire domain of IIEF-15 in the study groups. Notes: LLLT—low-level laser therapy, TENS—transcutaneous electrical nerve stimulation, #—*p* > 0.05 and **—*p* ≤ 0.01. The black lines highlight the significant differences between the values in the connected columns.

Initially, desire for sexual activity decreased in studied groups on average by 3.52 ± 1.12 points, without significant differences across groups (F = 0.60, *p*-value = 0.62). After treatment, no significant dynamics were registered in the control group (Figure 7). This aspect of sexual function, on the other hand, improved significantly in the LLLT and TENS groups, by 72.4% (*t* = 6.11, *p*-value = 0.0001, Cohen’s d = 2.09) and 50% (*t* = 4.26, *p*-value = 0.0002, Cohen’s d = 1.46), respectively. Compared with TENS, LLLT increased sexual desire by 11.7% (*t* = 1.63, *p*-value = 0.11, Cohen’s d = 0.56). However, this difference between groups was not statistically significant. A more pronounced improvement in sexual desire was observed with the combination of LLLT and TENS, with an average of 30.9% (*t* = 4.64, *p*-value = 0.0001, Cohen’s d = 1.60). Post-treatment outcomes in all treatment groups remained at the same level during the observation period, with no apparent deterioration.

#### 3.2.3. Sexual Satisfaction

Sexual satisfaction was assessed using two domains of the IIEF-15: intercourse satisfaction and overall satisfaction.

##### Intercourse Satisfaction

Before treatment, the mean intercourse satisfaction score was 5.74 ± 1.9 points, with no significant differences between groups (F = 0.359, *p*-value = 0.782). The improvement in intercourse satisfaction was 26.8% after LLLT (*t* = 2.42, *p*-value = 0.02 Cohen’s d = 0.85), 39.9% after TENS (*t* = 3.45, *p*-value = 0.0016, Cohen’s d = 1.16), and 56.4% after the combined use of LLLT and TENS (*t* = 4.80, *p*-value = 0.0001, Cohen’s d = 1.69).

In a comparative analysis, it can be noted that the level of intercourse satisfaction in the group of patients who underwent TENS is significantly higher than in patients after LLLT, by 11.7% (*t* = 2.06, *p*-value = 0.048, Cohen’s d = 0.69). It can also be noted that the combined use of LLLT and TENS increases the intercourse satisfaction after TENS by 8.5%, but these results did not reach the level of reliability (*t* = 1.57, *p*-value = 0.123, Cohen’s d = 0.69).

**Figure 8:**
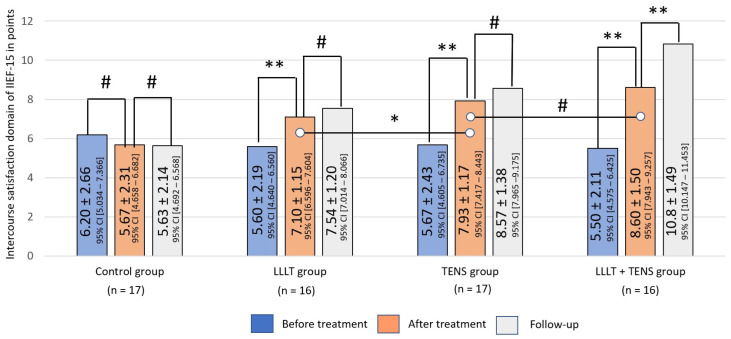
Dynamics of intercourse satisfaction of IIEF-15 in the study groups. Notes—LLLT—low-level laser therapy, TENS—transcutaneous electrical nerve stimulation, #—*p* > 0.05, *—*p* ≤ 0.05, and **—*p* ≤ 0.01. The black lines highlight the significant differences between the values in the connected columns.

By the end of the follow-up, the level of intercourse satisfaction was maintained without negative dynamics in the LLLT and TENS groups. With the combined use of LLLT and TENS, persistent positive dynamics was recorded, with an increase in the level of intercourse satisfaction at the post-treatment level by 25.9% (*t* = 4.16, *p*-value = 0.0001, Cohen’s d = 0.52). In the control group, no dynamics were detected during the post-treatment period and subsequent observation (Figure 8).

##### Overall Satisfaction

Across the study groups, the level of overall satisfaction reported by participants was uniformly low, averaging 2.99 ± 0.90 points (F = 0.406, *p*-value = 0.749).

**Figure 9:**
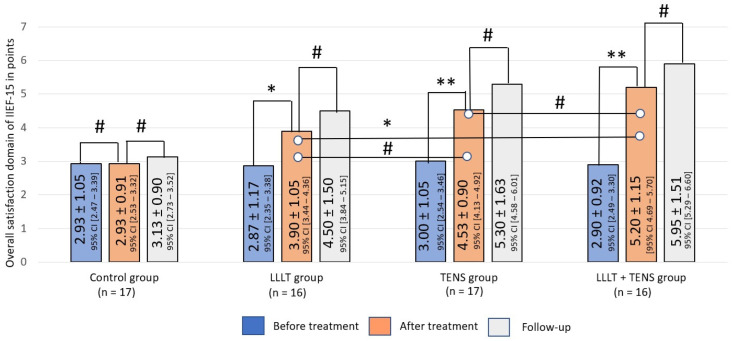
Dynamics of overall satisfaction of IIEF-15 in the study groups. Notes—LLLT—low-level laser therapy, TENS—transcutaneous electrical nerve stimulation, #—*p* > 0.05, *—*p* ≤ 0.05, and **—*p* ≤ 0.01. The black lines highlight the significant differences between the values in the connected columns.

Similarly, the post-treatment dynamics of overall satisfaction were significant higher after LLLT, by 35.8% (*t* = 2.60, *p*-value = 0.013, Cohen’s d = 0.92), after TENS by 51.0% (*t* = 4.43, *p*-value = 0.0001, Cohen’s d = 1.56), and after the combination of LLLT and TENS by 79.3% (*t* = 6.24, *p*-value = 0.0001, Cohen’s d = 2.1). In a comparative analysis, overall satisfaction after treatment was 15.4% higher (*t* = 1.80, *p*-value = 0.08, Cohen’s d = 0.61) with TENS than with LLLT. However, this superiority was not significant. The combined use of TENS was more effective in increasing overall satisfaction compared to the use of LLLT alone, by 33.3% (*t* = 3.33, *p*-value = 0.002, Cohen’s d = 1.18). In the follow-up, post-treatment levels remained constant for 3 months after the discontinuation of treatment. Treatment outcomes in the control group were not significantly different from baseline (Figure 9).

### 3.3. Exploratory Clinical Outcomes

#### Electromyography Examination

Needle EMG of the pelvic floor muscles revealed signs of denervation–reinnervation changes, of varying severity, in 38 patients. However, the mean values of the polyphasicity, duration, and amplitude of motor unit potentials in the studied groups were too different and not comparable (Table 2). However, the results of this examination were used to determine the presence and extent of damage to the motor fibers of the pudendal nerve.

The follow-up EMG examination was conducted in only 14 patients. The results showed positive dynamics in the treatment groups (n = 12) and no significant dynamics in the control group (n = 2) (Table 3). As a result of treatment, a significant increase in the number of polyphasic MUPs of 21% (*t* = 2.20, *p*-value = 0.03, Cohen’s d = 0.90) was noted, as well as an increase in MUPs with increased duration and amplitude of 41.6% (*t* = 3.56, *p*-value = 0.001, Cohen’s d = 1.45) and 59.8% (*t* = 3.34, *p*-value = 0.003, Cohen’s d = 1.36), respectively.

### 3.4. Side Effects

No side effects were observed after LLLT and TENS procedures.

## 4. Discussion

In this study, the efficacy of TENS and LLLT in the treatment of long-term ED after COVID-19 was investigated. Pathogenetic mechanisms of this disease may include neurogenic [80], psychogenic [81], vasculogenic [82], endothelial [17,79], and endocrine factors [83], both separately and in combination. In the acute period of COVID-19, hypoxia and multiple organ pathology play a significant role, which can be the cause of temporary ED [84].

Typically, the symptoms and complications of COVID-19 gradually regress after the virus is eliminated and immunity develops [84]. However, in many cases, the tissue changes are irreversible and persistent, leading to long-term ED [80,85,86]. These are the patients included in our study. Thus, in our study, ED is one type of long COVID-19.

In our other works, we noted that post-COVID-19 ED most often develops due to endothelial pathology and neuropathy of the pudendal nerve [80]. The effectiveness of LLLT in the treatment of endothelial pathology has been proven in many studies [64,80,85,86], and many works have demonstrated the recovery and protective effect of TENS in the treatment of various forms of neuropathy [67,68,70,71,72]. In this regard, we decided to assess and compare the efficacy of LLLT and TENS in the treatment of this disease. The study did not include patients with mental, vascular, and endocrine disorders, but only patients with endothelial and neurogenic disorders.

### 4.1. Erectile Function

This study demonstrated that TENS and LLLT both encourage ED regression. But after LLLT, the regression of ED was only 8.2% points higher than after TENS (*t* = 2.03, *p*-value = 0.049, Cohen’s d = 0.64).

The effectiveness of LLLT in the treatment of erectile dysfunction has been demonstrated in many experimental and clinical studies [80,85]. LLLT activates cytochrome C oxidase, a key enzyme in mitochondria. This activation increases the production of adenosine triphosphate (ATP), which is the primary energy source for cellular processes [87]. Another key pathogenic mechanism of LLLT is its antioxidant and anti-inflammatory effects, which are achieved by modulation of the routes of reactive oxygen species (ROS) and glutathione peroxidase (GSH-Px) [88,89,90]. In addition, LLLT activates signaling pathways, such as PI3K/Akt and MAPK/ERK, stimulating tissue regenerative processes [91,92,93]. LLLT has been shown to increase the concentration of calcium ions released from the endoplasmic reticulum and to activate intracellular Ca^2+^-dependent reactions [94]. Increased calcium levels activate endothelial nitric oxide synthase, which results in the production of NO [95]. NO relaxes vascular smooth muscle by activating soluble guanylate cyclase [96], resulting in vasodilation, increased blood flow, and filling of the corpora cavernosa [97]. Additionally, NO is important and critical for regulating vascular tone [97], controlling blood flow [98], promoting relaxation [80,99], and influencing cell growth and extracellular matrix production [100].

The effectiveness of TENS in the treatment of erectile dysfunction is associated with the functional and morphological improvement of the affected pudendal nerve [101,102]. In addition, TENS leads to the activation of parasympathetic fibers innervating the corpora cavernosa [102,103]. Sexual intercourse is a very complex process in which sensory function plays an important role [104]. The final result of the therapeutic effect of TENS is a combined improvement in both reflexogenic and psychogenic erections as a result of the simultaneous excitation of the sacral spinal cord at the S2–S4 level [105,106] and the primary somatosensory cortex [102]. In addition, the stimulation of the pudendal nerve contributes to an increase in penile rigidity due to the improvement of muscle tension in ischiocavernosus and bulbocavernosus muscles of the pelvic floor [103]. The increased pressure of the contracted muscles compresses the deep veins that collect blood from the corpora cavernosa, reducing the outflow and effectively creating a closed vascular system that maintains high pressure in the corpora cavernosa [107,108]. In addition, the electrical stimulation of parasympathetic nerves and alpha adrenergic receptors triggers the release of neurogenic NO from nerve endings, which then activates endothelial cells to produce endothelial NO [109,110,111]. It is important to note that the role of TENS in the synthesis and accumulation of growth factors in the application area has been proven in a number of experimental studies [112,113]. Furthermore, the anxiolytic and anti-depressive effect of TENS, caused by the release of central endorphins, and normalization of hippocampal function and hemispheric connections, can have a positive effect on erectile function [66,114,115,116]. In many cases primary ED is complicated by secondary anxiety–depressive disorders, which negatively affect ED [117].

### 4.2. Orgasmic Function

Both TENS and LLLT significantly improved orgasmic function, and neither treatment was found to be superior to the other by the end of the treatment or during the follow-up period.

It is important to note that erection and orgasm are separate but interrelated aspects of male sexuality [118]. On the one hand, sustaining and maintaining an erection throughout intercourse undoubtedly facilitates the process of achieving orgasm and improves its quality [119]. However, on the other hand, erectile dysfunction is not an obstacle to achieving orgasm. Alternatively, orgasm can be achieved through non-penetrative sexual acts such as oral sex, genital caresses, or during masturbation [120]. Conversely, with some diseases, the patient may experience orgasmic dysfunction, delayed orgasm, and anorgasmia, despite a stable erection [121]. Thus, both processes involve complex neurological and physiological pathways, but disturbances in either function can occur independently. The effectiveness of LLLT in improving orgasmic function has been described in rare studies and is generally secondary in nature [101,122]. The effect of TENS on orgasmic function in men has been studied with approximately the same rarity [123]. It should be noted here that the obtained correlation data indicate that the improvement in orgasmic function was associated with an improvement in erection on one side and with an improvement in sensory function in the genital area.

### 4.3. Sexual Desire

Sexual desire, or libido, and erection are related but distinct. The level of libido is significantly influenced by both male hormone levels, particularly testosterone, and a person’s psychological state. Low testosterone levels can lead to a reduced desire for sex [124], while psychological factors such as stress, depression, and relationship problems can also negatively impact libido [125]. More often than not, the inability to achieve or maintain an erection can impact confidence levels and lead to anxiety about sex, which in turn can reduce overall desire [126]. The findings of our study demonstrated that TENS and LLLT were highly effective in increasing libido in the majority of patients. Although there was a greater increase in libido following LLLT by 11.7% compared to TENS, the small sample size made the differences between groups non-significant (*t* = 1.63, *p*-value = 0.11, Cohen’s d = 0.56).

Other studies have demonstrated a regression of clinical symptoms of infertility alongside a simultaneous improvement in libido after the use of LLLT [127]. The obtained results indicate that the improvement in libido in these studies is of endocrine origin. However, it is difficult to confirm unequivocally that the improvement in libido in our patients is due to this reason, since all patients included in our study did not have endocrine disorders. In our opinion, the improvement in libido after LLLT and TENS has a secondary psychological basis and is associated mainly with improved erection, which gives the patient greater confidence during sexual intercourse. Notably, improved genital sensation following TENS significantly increases somatosensory afferentation, resulting in higher patient satisfaction and a greater desire for sexual activity.

### 4.4. Sexual Satisfaction

Undoubtedly, the final and most important outcome of sexual relations and sexual intercourse is sexual satisfaction. According to the results of our study, a significant improvement in sexual satisfaction was registered after using TENS and LLLT, with an 11.7% (*t* = 2.06, *p*-value = 0.048, Cohen’s d = 0.69) advantage for TENS when considering intercourse satisfaction and a 15.4% (*t* = 1.80, *p*-value = 0.08, Cohen’s d = 0.61) advantage when considering overall satisfaction. The advantage of TENS over LLLT is explained by the multifaceted action of TENS, which, in addition to a positive effect on erection, has an anxiolytic [66] and neurogenic recovery effect [67,70,71,72].

### 4.5. Prolonged Effect of LLLT and TENS

During the 3-month follow-up, the post-treatment results regarding erectile function, orgasmic function, sexual desire, and sexual satisfaction were maintained without negative dynamics. Moreover, a tendency toward improvement was observed. However, these changes did not reach the level of reliability. A study of the literature has shown that the prolonged effect of LLLT has been proven experimentally and clinically [128,129]. A prolonged effect was also noted 3–6 months after the end of TENS treatment of various pathologies of the peripheral and central nervous system [67,70,71,72]. The reason for the prolonged effect of LLLT is, first of all, the acceleration of regenerative processes in tissues, with an increase in their resistance to ischemia and apoptosis [130,131,132]. The prolonged effect of TENS is associated with a reduction in degenerative changes, and improvement in regenerative changes, due to the activation of compensatory reactions in neighboring nerve fibers and associative central structures. This can manifest as improved nerve fiber density, increased growth factor secretion, enhanced blood flow, and a decrease in pro-inflammatory cytokines, which collectively support nerve recovery [68,70].

### 4.6. Combined Use of LLLT and TENS

The idea to combine LLLT and TENS in the treatment of patients with post-COVID-19 ED arose due to the different therapeutic mechanisms of these two effective methods. The outcomes were as expected. The combined use of LLLT and TENS was more effective than the separate use of LLLT and TENS. Erectile function turned to be stronger by 20% (*t* = 3.16, *p*-value = 0.003, Cohen’s d = 1.05) in TENS compared to LLLT results, and tactile sensation was better by 25% (*t* = 2.032, *p*-value = 0.0491, Cohen’s d = 0.64) in LLLT compared to TENS therapy. It can also be noted the superiority of the combined use of LLLT and TENS in improving orgasmic function, by 30.5% (*t* = 3.38, *p*-value = 0.002, Cohen’s d = 1.12), in improving sexual desire, by 30.9% (*t* = 4.64, *p*-value = 0.0001, Cohen’s d = 1.60), and in improving overall sexual satisfaction, by 33.3% (*t* = 3.33, *p*-value = 0.002, Cohen’s d = 1.18), compared to LLLT results. The high efficiency of this method is due to the expansion of the spectrum of therapeutic effects, on the one hand, and the enhancement of the TENS effect after LLLT, on the other hand.

### 4.7. Dynamics of Electromyography Changes

After treatment, patients who received active LLLT and TENS therapy showed signs of an increased reinnervation process, which manifested by increasing polyphasic MUPs and inhaling MUPs with greater duration and amplitude. However, all patients in all treatment groups were merged into one because of the small sample size. As a result, our study was unable to compare the improvement in neurophysiological parameters following treatment between the LLLT and TENS groups.

### 4.8. Endothelial Sensing and Signaling

**Figure 10:**
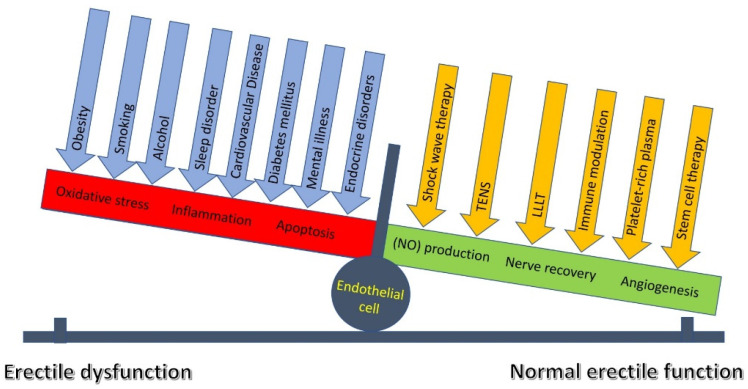
External triggers of activation and deactivation of endothelial cells. Notes: LLLT: low-level laser therapy, and TENS: transcutaneous electrical nerve stimulation. Blue arrows: factors negatively affecting endothelial cell function; orange arrows: factors positively affecting endothelial cell function. The red column reflects degenerative changes in the endothelial cell, and the green column reflects regenerative processes in the endothelial cell.

Overall, LLLT and TENS are effective in the treatment of ED, improving pudendal nerve activity and endothelial function. However, factors like smoking [133] and excessive alcohol use [134] negatively impact endothelial cells, complicating treatment by creating an unfavorable environment for recovery. The crucial pathogenetic mechanism of smoking and alcohol use is an imbalance between free radicals and antioxidants, leading to oxidative stress. This stress damages cellular components like DNA, lipids, and proteins, and triggers chronic inflammation. Sequentially, this process reduces the release and accumulation of NO, leads to cell damage [99,135], and, in severe cases, to apoptosis [136]. In addition, comorbidities, such as cardiovascular diseases [137], endocrine disorders [138,139], obesity [140], insomnia [136], and mental illness [117,141] may be the underlying cause of erectile dysfunction or the reason for the patient’s reduced response to treatment. Probably, the combined use of LLLT and TENS with regenerative and molecular therapies could achieve the maximum effect due to the increase in NO production, acceleration of angiogenesis, and stimulation of nerve regeneration (Figure 10). To address this issue, further research is needed in the future, using a combination therapy of LLLT and TENS with regenerative and molecular therapies such as platelet-rich plasma, stem cell therapy, and immunomodulation. In accordance with the above, personalized ED treatment algorithms should be created through a thorough assessment of all negative factors and comorbid backgrounds, using the most effective pharmacological and non-pharmacological interventions in accordance with the etiology and severity of ED.

## 5. Limitations

This study is not without limitations. The first limitation was that sham TENS was performed with minimal sensory input, threshold amplitude, and low frequency, which is not effective and, at the same time, raises doubts in patients’ minds about the effectiveness of the treatment. Additionally, to exclude the therapeutic effect of this current, the electrodes were fixed in the lower abdomen, further from the innervation zone of the pudendal nerve. Another limitation was the use of a relatively subjective method of determining penile tactile sensation, using a ten-point scale. Yet another potential limitation was that not all patients could undergo post-treatment electromyography. In addition, the baseline results of these examinations were not comparable. In this regard, the obtained results of the EMG study cannot be used to determine the effectiveness of a particular treatment method and most likely serve as a basis for further research in this area. However, the main value of this section is the study of the patterns of pudendal nerve damage in the studied patients before treatment, while a comparative analysis between groups is not required. This study may also be limited by the statistical analysis of secondary outcome measures, which used data transformations, normalization, and outlier limitation to adjust for variations in baseline values among groups. Further shortcomings include the inability to assess the link between COVID-19 severity and the severity of ED, as well as ED treatment response. This lack of information stemmed from patients’ COVID-19 infections occurring outside the study setting (in other hospitals or at home), preventing the collection of detailed data on the clinical course of the illness.

## 6. Conclusions

According to IIEF-15 dynamics, LLLT and TENS are both very beneficial in the treatment of erectile dysfunction following COVID-19, with LLLT showing a moderately better outcome than TENS. LLLT and TENS were found to have significant positive therapeutic effects on orgasm, sexual desire, and sexual satisfaction, among other aspects of sexual function. Nevertheless, the combination of LLLT and TENS proved to be much more successful in enhancing all IIEF domains, expanding the therapeutic effect spectrum, and improving the TENS effect following LLLT application. Only after TENS did genital hypoesthesia reliably regress, and the effect was amplified when TENS and LLLT were combined. Further studies are recommended to examine the effectiveness of LLLT and TENS in combination with regenerative therapies and conventional pharmacological treatments, taking into account a personalized approach.

## Figures and Tables

**Table 1:** Characteristics of the participants.

Characteristic	Control Group	LLLT Group	TENS Group	LLLT + TENS Group	F *	*p* **
Number of the participants, n	20	21	21	20		
Age, years	41.2 ± 7.7	41.2 ± 6.9	41.8 ± 6.5	41.5 ± 6.0	0.04	0.99
Disease duration, months	8.70 ± 1.9	8.90 ± 1.8	8.80 ± 1.6	8.60 ± 1.6	0.11	0.95
Duration of completed pharmacotherapy, months	2.53 ± 0.77	2.50 ± 0.63	2.70 ± 0.65	2.63 ± 0.67	0.37	0.78
The erectile function domain, points	16.3 ± 3.75	15.3 ± 3.51	15.4 ± 3.25	16.5 ± 3.36	0.22	0.87
Hypoesthesia, points	3.60 ± 0.92	3.83 ± 1.23	3.90 ± 1.12	3.83 ± 0.91	0.32	0.99

Note: TENS—transcutaneous electrical nerve stimulation; LLLT—low-level laser therapy; *p*—statistical significance; mean ± standard deviation (SD); F *—F-value by ANOVA test; and *p* **—*p*-value by ANOVA test.

**Table 2:** Characteristics of registered denervated motor unit potentials.

Characteristic	Control Group	LLLT Group	TENS Group	LLLT + TENS Group	F *	*p* **
Number of the participants, n	9	8	13	8		
Polyphasic MUP (%)	46.1 ± 10.5	39.4 ± 18.6	38.8 ± 10.4	24.4 ± 10.5	4.364	0.011
Minimum (%)	30	15	20	15		
Maximum (%)	65	50	55	45		
Median (%)	45	45	40	20		
Increase in MUP latency (%)	37.8 ± 8.08	29.4 ± 12.4	23.6 ± 7.53	29.4 ± 10.2	4.041	0.015
Minimum (%)	25	15	10	15		
Maximum (%)	50	50	35	45		
Median (%)	35	27.5	25	27.5		
Increase in MUP amplitude	11.7 ± 4.08	8.75 ± 5.99	15.0 ± 4.11	11.9 ± 5.21	2.917	0.048
Minimum (%)	5	0	10	0		
Maximum (%)	20	15	20	20		
Median (%)	10	10	15	12.5		

Note: TENS—transcutaneous electrical nerve stimulation; LLLT—low-level laser therapy, *p*—statistical significance, mean ± standard deviation (SD), F *—F-static by ANOVA test, and *p* **—*p*-value by ANOVA test.

**Table 3:** Characteristics of registered denervated motor unit potentials in treatment groups.

Characteristic	Before Treatment	After Treatment	*t* *	*p* **	Cohen’s d
Number of the participants, n	12	12			
Polyphasic MUP (%)	33.8 ± 7.42	41.0 ± 8.51	2.20	0.03	0.90
Minimum (%)	25	25			
Maximum (%)	50	55			
Median (%)	35	37.5			
Increase in MUP latency (%)	25.0 ± 7.38	35.4 ± 6.89	3.56	0.001	1.45
Minimum (%)	15	20			
Maximum (%)	40	45			
Median (%)	25	35			
Increase in MUP amplitude	11.7 ± 4.08	18.7 ± 5.99	3.34	0.003	1.36
Minimum (%)	10	0			
Maximum (%)	20	25			
Median (%)	12.5	17.5			

Note: TENS—transcutaneous electrical nerve stimulation; LLLT—low-level laser therapy *p*—statistical significance, Mean ± Standard deviation (SD), *t* *—*t*-statistic and *p* **—*p*-value.

## Data Availability

The original contributions presented in this study are included in the article. Further inquiries can be directed to the corresponding authors.
